# Gastric heterotopic pancreas and stromal tumors smaller than 3 cm in diameter: clinical and computed tomography findings

**DOI:** 10.1186/s40644-018-0161-9

**Published:** 2018-08-07

**Authors:** Li-ming Li, Lei-yu Feng, Xiao-hua Chen, Pan Liang, Jing Li, Jian-bo Gao

**Affiliations:** 1 Department of Radiology, The First Affiliated Hospital of Zhengzhou University, Zhengzhou, 450052 Henan Province China; 2 Department of Internal Medicine, The First Affiliated Hospital of Zhengzhou University, Zhengzhou, 450052 Henan Province China

**Keywords:** Gastric, Heterotopic pancreas, Stromal tumor, Computed tomography

## Abstract

**Background:**

Identifying gastric heterotopic pancreas and stromal tumors is difficult. Few studies have reported computed tomography (CT) findings for differentiating lesions less than 3 cm in diameter. In this study, we aimed to identify clinical characteristics and CT findings that can differentiate gastric heterotopic pancreatic lesions from stromal tumors less than 3 cm in diameter.

**Methods:**

A total of 132 patients with pathologically confirmed gastric heterotopic pancreas (*n* = 66) and stromal tumors (*n* = 66) were included. Each group was divided into primary (*n* = 50) and validation cohort (*n* = 16). Clinical characteristics and CT findings were retrospectively reviewed. CT findings included location, border, contour, growth pattern, enhancement pattern and grade, the enhancement value of tumor, enhancement ratio of tumor, and enhancement ratio of tumor to pancreas in venous phase. The findings in the two groups were compared using the Pearson χ^2^ test or Student t-test. Receiver operating characteristic curves were used to determine areas under the curve and optimal cut-offs.

**Results:**

Significant differences were observed between heterotopic pancreas and stromal tumors in the distribution of tumor location, border, contour (all *P* < 0.001), enhancement values (*P* < 0.001), enhancement ratios of tumors (*P* < 0.001), and enhancement ratios of tumors to pancreas (*P* < 0.001). No significant differences existed in growth pattern (*P* = 0.203). The area under the curve differed significantly between enhancement ratio of tumor to pancreas and enhancement ratio (*P* = 0.030). There were significant differences in above characteristics between two groups in validation cohort.

**Conclusions:**

Heterotopic pancreas has characteristic CT features differentiating it from stromal tumors.

## Background

Heterotopic pancreatic (HP) masses and stromal tumors (STs) are common gastric submucosa tumors. HP masses are typically found in autopsy or surgery, during which the frequency is approximately 0.2 to 0.25% [1, 2]. Approximately 10–15 per million people worldwide are diagnosed with gastrointestinal STs each year, with most of these tumors located in the stomach [3]. Both the management and prognosis of these two tumors are different [4–6]. STs are aggressive tumors with a potential tendency for malignancy, and the risk increases as the tumor increases in size. They require resection once detected, and occasionally, chemotherapy is required in cases of metastasis [7]. HP is a congenital anomaly, similar to hamartoma. Most patients are recommended to undergo surveillance because HP is generally asymptomatic, and only a few patients need to be treated because of complications [8]. In view of the above, an accurate preoperative diagnosis of submucosal tumors is critical.

Identifying HP masses and STs only by clinical features is difficult because both can manifest as abdominal pain, abdominal distension, and other symptoms. Although gastroscopic biopsy is regarded as the gold standard for the diagnosis of tumors, limitations include its invasive nature, sampling errors, diagnostic errors, long waiting times for immunohistochemistry results [9], and powerless assessing situations outside the tumor. Computed tomography (CT), as a common imaging examination method, has been used in the preoperative evaluation of submucosal tumors, and can be used as a supplemental tool in differentiating HP masses from STs [10].

Although many studies have been conducted on the imaging features of HP masses and gastric stromal tumors (STs), most have employed endoscopic ultrasonography [11, 12], with few adopting CT characteristics, and most have been case reports [10, 13]. To our knowledge, no studies have reported CT findings for differentiating gastric submucosal tumors less than 3 cm in diameter. Kim [14] reviewed CT findings of HP masses and other gastric submucosal tumors smaller than 4 cm, but size differences between the two groups inevitably led to errors in the results. Therefore, the purpose of this study was to analyze the clinical characteristics and CT findings of HP masses and STs less than 3 cm in diameter, identified in our hospital within a 5-year period, and to identify the features that differentiate one from the other.

## Methods

This retrospective study was approved by the Institutional Review Board of the First Affiliated Hospital of Zhengzhou University, and the requirement for informed consent was waived.

### Patients

**Fig. 1:**
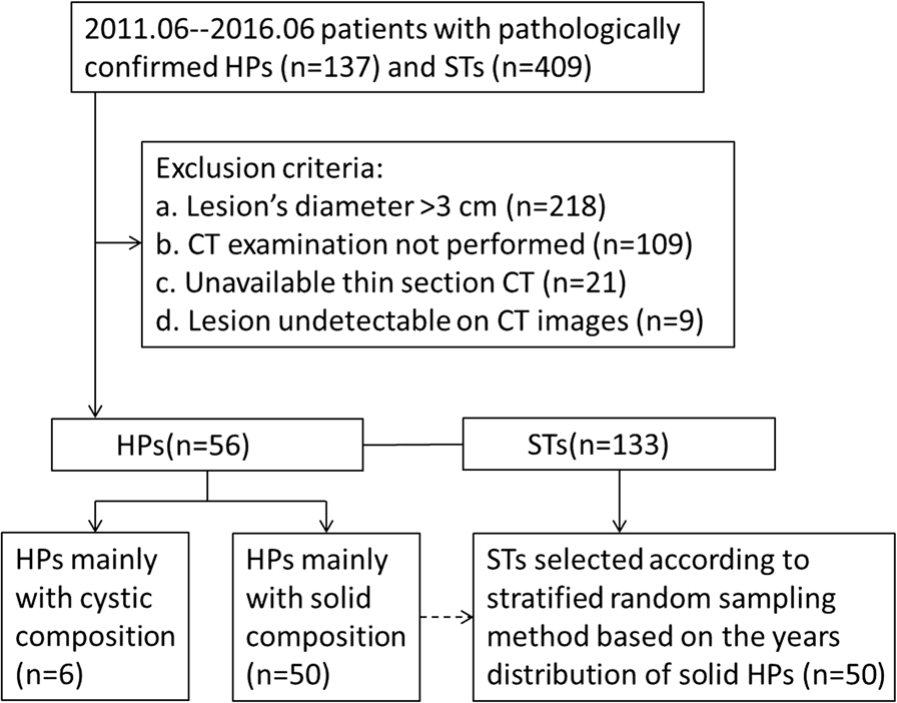
Flowchart illustrating patients enrolled in the study. HP: Heterotopic pancreas, STs: Stromal tumors

A total of 137 patients with HP masses and 409 patients with STs in primary cohort, pathologically confirmed between June 2011 and June 2016, were selected from our hospital database. Among them, patients who fulfilled the following criteria were included: (1) available dual-phase contrast–enhanced CT images; (2) available thin-layer images; (3) lesions less than 3 cm in diameter; (4) lesions detectable on CT images; (5) lesions with a mainly solid composition. Lesions mainly with cystic components were excluded because of their particular CT findings and easy to distinguish with gastric stromal tumors [15–17]. A total of 189 patients met the inclusion criteria; however, numbers differed between the two groups, and therefore, the same numbers of STs were selected according to stratified random sampling method, based on the yearly distribution of HP masses. Finally, 100 patients (HP masses = 50, GISTs = 50) comprised our study population. A total of 32 patients (HP = 16, ST = 16) in validation cohort, pathologically confirmed between July 2016 and January 2018, were included from our hospital database using the same criterion to primary cohort. A flowchart is presented in Fig. 1.

### CT image acquisition

CT images were obtained using a 16-channel multi-detector CT scanner (Brilliance 16, Philips Medical Systems, Cleveland, OH, USA) or a 64-channel multi-detector CT scanner (Discovery CT750 HD CT Scanner, GE Healthcare Milwaukee, WI, USA). The parameters of the Brilliance 16 scanner were as follows: detector collimation, 1.5 mm; pitch, 1.25:1; tube voltage, 120 kVp; tube current, 80–270 mAs; rotation time, 0.6 s. The parameters of the Discovery CT750 scanner were as follows: detector collimation, 0.625 mm; pitch, 1.375:1; tube voltage, 120 kVp; tube current, 80–270 mAs; rotation time, 0.5 s. For the contrast-enhanced CT study, 80–90 mL of 350 or 370 mg I/mL iodinated contrast agent was injected via a peripheral vein at a flow rate of 3.0–3.5 mL/s, using a dual high-pressure syringe. Dual-phase contrast–enhanced CT images were obtained by scanning the images 10 s and 50–65 s after attenuation of the descending thoracic aorta reached 100 Hounsfield units, using the bolus-tracking technique, for the arterial and venous phases, respectively. Axial, coronal, and sagittal CT images were reconstructed with a 3-mm section thickness and a 3-mm reconstruction interval at an Application Development Workstation (Advantage Windows 4.4; GE Medical Systems, Chicago, IL, USA).

### Clinical and image analysis

A clinical attending physician (L.F.) with 5 years of experience retrospectively reviewed the clinical data, including age, sex, chief complaint, and duration of symptoms. Chief complaints were classified as gastric pain gastric pain, abdominal distension, and other symptoms, including lesions found through physical examination [18]. Duration time of symptoms was divided into ≤6 months and > 6 months.

### Qualitative analysis

**Fig. 2:**
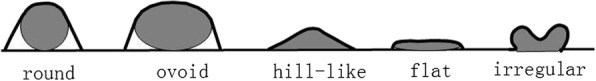
Contours of tumors. Tumorous contours were classified as round, ovoid, hill-like, flat or irregular in shape

Two radiologists (J.G. and P.L., with 25 and 6 years of experience, respectively), who were blinded to the pathological results, analyzed the CT images by consensus. We analyzed the following CT findings: location, lesion border, contour, growth pattern, enhancement pattern, peak enhancement phase and enhancement grade, presence of prominent thickness and enhancement of overlying mucosa, presence of central umbilication, and presence of calcification, ulceration, and multiple lesions. The stomach location is anatomically divided into three portions, the upper (U), middle (M), and lower (L) parts, by the lines connecting the trisected points on the lesser and greater curvatures according to Japanese classification of gastric carcinoma: 3rd English edition [19]. Tumorous contours were classified as flat, hill-like, ovoid, round, or irregular in shape [14] (Fig. 2). Growth patterns were classified as exo-luminal, endo-luminal, or mixed. Enhancement pattern was classified as homogeneous or heterogeneous. Enhancement grade was classified as mild, moderate, or marked.

### Objective analysis

Two radiologists (J.L. and X.C., both with 5 years of experience), who were aware of the gastric lesions but were blinded to the pathological results, measured the lesion diameter and CT attenuation. The CT attenuation values of the pancreas and tumors in the venous phase and plain phase were measured in Hounsfield units by two radiologists. The averages were then used to calculate the enhancement value of tumor (HU venous - HU plain), the enhancement ratio of tumor (HU venous- HU plain / HU plain) and the enhancement ratio of tumor to pancreas in venous phase (HU tumor / HU pancreas). The region of interest (ROI) ranging from 9 mm^2^ to 30 mm^2^ was placed to encompass the strongest enhancing portion and to avoid necrosis and calcification. The long diameter (LD) and short diameter (SD) were measured by the two radiologists. The averages were then used to calculate the LD/SD ratio.

### Statistical analysis

All statistical calculations were performed using the Statistical Package for the Social Sciences (SPSS 21.0, Chicago, IL, USA), and a *P*-value of less than 0.05 was considered to be statistically significant. Categorical variables including clinical characteristics (sex, chief complaint, duration of symptoms) and qualitative CT features (e.g., location, lesion border, contour, growth pattern, enhancement pattern, and enhancement grade) were described as frequencies or percentages. The Pearson χ^2^ tests (including continuity correction) or Fisher’s exact test were used to evaluate the differences between the two groups. Continuous variables subjected to a normality test were reported as means and standard deviation and were compared using Student’s t-tests (including the correct t-test).

Receiver operating characteristic (ROC) curves of the LD/SD ratio, the enhancement value, the enhancement ratio of tumor, the enhancement ratio of tumor to pancreas were obtained to generate the area under the curve (AUC) and an optimal cut-off, where the sum of the sensitivity and the specificity was the maximum. In general, an AUC between 0.5 and 0.7 suggests low diagnostic value, an AUC between 0.7 and 0.9 suggests medium diagnostic value, and an AUC between 0.9 and 1 suggests high diagnostic value. Sensitivity, specificity, and odds ratio (ORs) with 95% confidence intervals (CIs) were analyzed for variables that differed significantly between the two groups. Variables with significant differences in primary cohort were compared in validation cohort.

## Results

### Clinical analysis

Table 1 summarizes the clinical characteristics between the two groups. No significant differences were observed in sex, chief complaint, and duration of symptoms. However, age distribution differed significantly between the two groups (HP masses = 41.22 ± 11.76 y; STs = 59.18 ± 11.15 y, *P* < 0.001).

**Table 1:** Comparison of clinical characteristics between two lesions n (%)

Clinical characteristics	HP masses (*n* = 50)	STs (*n* = 50)	*P* Value
Age(year)	41.22 ± 11.76	59.18 ± 11.15	< 0.001^a^
Gender			0.684
Male	21 (42)	19 (38)	
Female	29 (58)	31 (62)	
Chief complaint			0.191
Gastric pain	27 (54)	18 (36)	
Distension	7 (14)	9 (18)	
Other	16 (32)	23 (46)	
Duration of symptoms			0.680
≤ 6 months	32 (64)	30 (60)	
> 6 months	18 (36)	20 (40)	

^a^*P* < 0.05. Calculated with Student’s t-test. *HP* Heterotopic pancreas, *STs* Stromal tumors

### Qualitative analysis

**Fig. 3:**
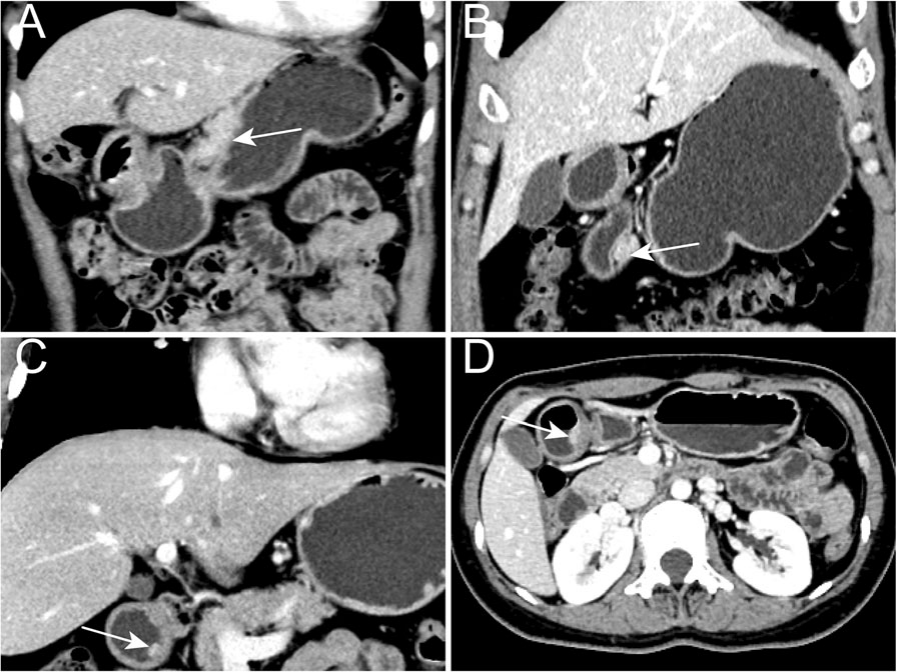
Representative CT images in the venous phase of heterotopic pancreatic masses (white arrows). **a** The coronal image shows an ill-defined irregular mass in the gastric middle body with exo-luminal growth pattern. **b** The coronal image shows an ill-defined ovoid mass in the gastric lower body with mixed growth pattern. **c** The coronal image shows a will-defined flat mass in the gastric lower body with endo-luminal growth pattern. **d** The axial image shows an ill-defined hill-like mass in the gastric lower body with endo-luminal growth pattern. The LD/SD ratio of this lesion is 1.85 (15.83/8.54 mm). The relative enhancement ratio of HP masses to the pancreas is 0.94 (101.25/107.58 HU)

**Fig. 4:**
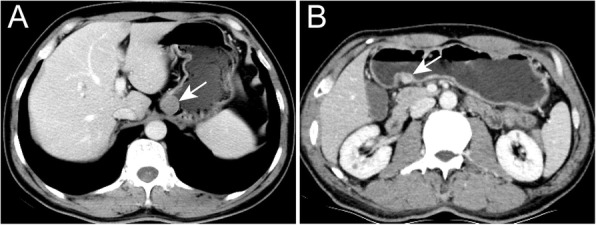
Representative CT images in the venous phase of stromal tumors (white arrows). **a** The axial image shows a will-defined round mass in the gastric upper body with endo-luminal growth pattern. The LD/SD ratio of this lesion is 1.12 (20.14/17.85 mm). The relative enhancement ratio of HP masses to the pancreas is 0.59 (56.87/96.81 HU). **b** The axial image shows an ill-defined round mass in the gastric lower body with exo-luminal growth pattern

**Fig. 5:**
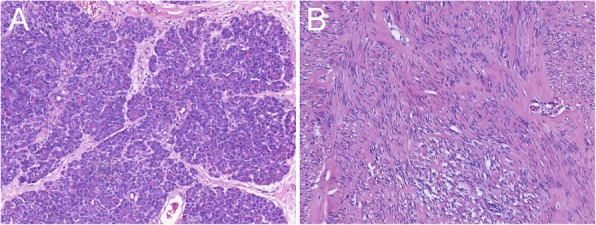
Photomicrographs of gastric submucosal tumors stained with Hematoxylin-eosin stain. **a** Heterotopic pancreatic mass is composed of pancreatic acini and ducts. **b** Stromal tumor is composed of homogenous spindle cells

Table 2 summarizes the morphologic qualitative CT findings. A significant difference was observed in the distribution of tumor location (*P* < 0.001); most HP masses (66%, 33/50) were located in the lower part, whereas most (76%, 38/50) STs were located in the upper part. With regard to lesion border, 28 (56%) of the 50 HP masses were ill defined and 45 (90%) of the 50 STs were well defined. HP masses showed diverse shapes, and were mainly (86%, 43/50) flat, hill-like, or ovoid; few (14%, 7/50) were round or irregular in shape. Most (88%, 44/50) STs were round or ovoid. Homogeneous enhancement in the venous phase was observed in most of the HP masses and STs, whereas marked enhancement was observed in most (62%, 31/50) HP masses and moderate enhancement were observed in most (58%, 29/50) STs. No statistical differences existed in growth pattern between the two groups (*P* = 0.203). The presence of prominent thickness and enhancement of the overlying mucosa, central umbilication, and multiple lesions was detected in only a few (10, 6, 2%) of the patients with HP masses, whereas the presence of calcification and ulceration was detected in only a few (10, 8%) of the patients with STs. Notably, two ectopic pancreatic lesions in one patient were found, and only the bigger lesion was included in our study because the other was too small to analyze. Representative images are presented in Figs. 3, 4, and 5.

**Table 2:** Comparison of subjective CT findings between two lesions n (%)

CT findings	HP masses (*n* = 50)	STs (*n* = 50)	*P* Value
Location			< 0.001^a^
The upper part	2 (4)	38 (76)	
The middle part	15 (30)	8 (16)	
The lower part	33 (66)	4 (8)	
Border			< 0.001^a^
Well defined	22 (44)	45 (90)	
Ill defined	28 (56)	5 (10)	
Contour			< 0.001^a^
Round	4 (8)	21 (42)	
Ovoid	24 (48)	23 (46)	
Hill-like	10 (20)	4 (8)	
Flat	10 (20)	1 (2)	
Irregular shape	3 (6)	1 (2)	
Growth pattern			0.203
Endo-luminal	38 (76)	35 (70)	
Exo-luminal	7 (14)	4 (8)	
Mixed	5 (10)	11 (22)	
Enhancement pattern			0.084
Homogeneous	46 (92)	40 (80)	
Heterogeneous	4 (8)	10 (20)	
Peak enhancement phase			0.236
Venous phase	43 (86)	48 (96)	
Arterial phase	2 (4)	0	
Both	5 (10)	2 (4)	
Enhancement grade			0.003^a^
Marked	31 (62)	14 (28)	
Moderate	15 (30)	29 (58)	
Mild	4 (8)	7 (14)	
Central umbilication	3 (6)	0	0.241
Prominent enhancement of overlying mucosa	5 (10)	0	0.066
Multiple lesions	1 (2)	0	0.500
Ulceration	0	4 (8)	0.126
Calcification	0	5 (10)	0.066

^a^*P* < 0.05. Calculated with χ^2^ test

### Quantitative analysis

Significant differences were observed in the variables relating to the lesion enhancement grade, including the enhancement value, the enhancement ratio, the enhancement ratio to pancreas between the two groups. HP masses had significantly higher enhancement value (HP masses = 43.54 HU ± 11.78, STs = 29.16 HU ± 13.69, *P* < 0.001), enhancement ratio (HP masses = 1.08 ± 0.45, STs = 0.77 ± 0.37, *P* < 0.001), and enhancement ratio to pancreas (HP masses = 0.93 ± 0.15, STs = 0.74 ± 0.16, *P* < 0.001) than STs.

No significant differences were found in LDs (HP masses = 15.08 mm ± 5.96, STs = 16.79 mm ± 5.96, *P* > 0.05), but significant differences were observed in SDs (HP masses = 10.29 mm ± 4.64, STs = 13.69 mm ± 5.27, *P* < 0.001) between the two groups. The mean LD/SD ratio for HP masses was significantly higher than that for STs (HP masses = 1.61 ± 0.61, STs = 1.26 ± 0.25, *P* < 0.001).

### Sensitivity and specificity analysis

**Fig. 6:**
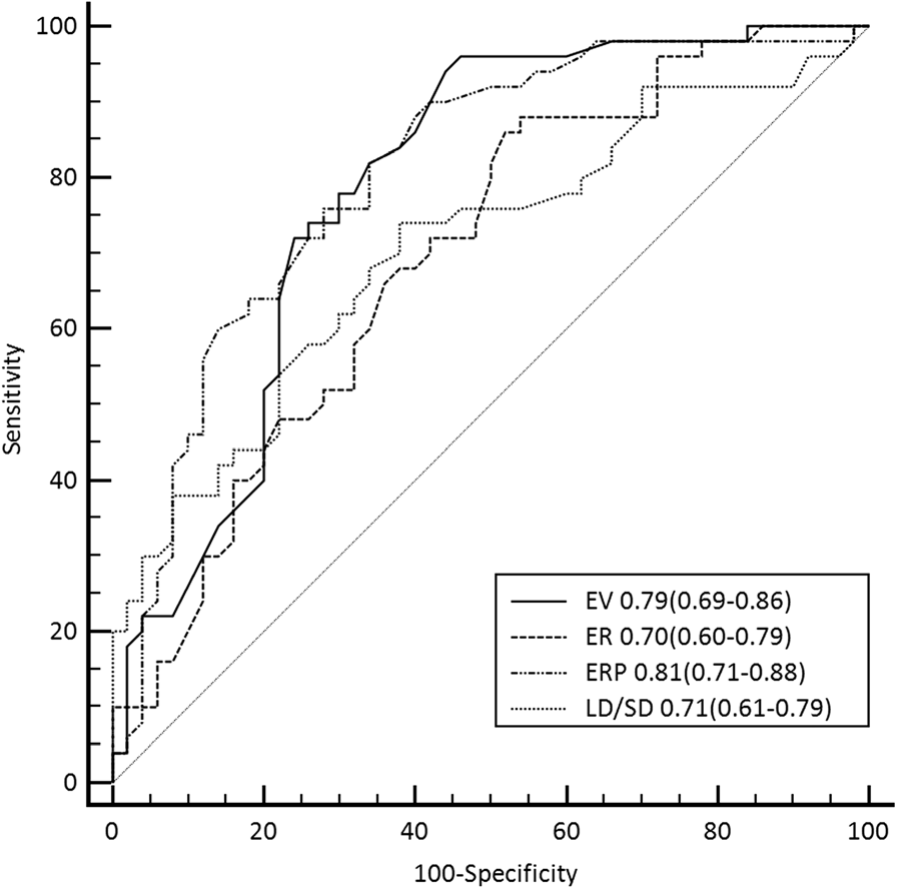
ROC curves for the enhancement value (EV), the enhancement ratio (ER), the enhancement ratio to pancreas (ERP), the LD/SD ratio in the differentiation of heterotopic pancreas from stromal tumors. Data are presented as area under the curve (95% CI). There were significant differences between the enhancement ratio to pancreas and the enhancement ratio, the enhancement value and the enhancement ratio

Using ROC analysis, cut-off values for the LD/SD ratio, the enhancement value, the enhancement ratio and the enhancement ratio to pancreas were set at 1.29, 27.50 HU, 0.66, and 0.72, respectively. The AUCs were 0.71, 0.786, 0.70, and 0.81, respectively (Fig. 6). The above continuous variables were transformed into categorical variables according to the cut-off values.

Table 3 summarizes the sensitivity, specificity, and OR of each significant variable, including location in the lower part, marked enhancement, flat and irregular shapes, ill-defined border, LD/SD > 1.29, the enhancement value > 27.50 HU, the enhancement ratio > 0.66, and the enhancement ratio to pancreas > 0.72.

**Table 3:** Sensitivity and specificity of CT findings in diagnosis of HP masses

CT findings	Sensitivity (%)	Specificity (%)	OR (95% CI)
Enhancement value > 27.50 HU	96 (48/50)	54 (27/50)	28.17 (8.59, 92.39)
Lower part	66 (33/50)	92 (46/50)	22.32 (8.06,61.48)
Enhancement ratio to pancreas > 0.72	92 (46/50)	56 (28/50)	14.64 (5.27, 40.68)
Ill-defined border	56 (28/50)	90 (45/50)	11.45 (3.89, 33.72)
Flat or hill-like	26 (32/50)	96 (48/50)	8.43 (0.64, 32.74)
Enhancement ratio > 0.66	88 (44/50)	46 (23/50)	6.25 (2.40, 16.29)
LD/SD ≥1.29	68 (34/50)	68 (34/50)	4.52 (1.98, 10.26)
Marked enhancement	62 (31/50)	72 (36/50)	4.20 (1.83, 9.58)

*OR* odds ratio, *CI* confidence interval, *LD* long diameter, *SD* short diameter

Variables in the validation cohort.

Clinical characteristics and CT findings in validation cohort are summarized in the Table 4. There were significant differences in all variables between two groups in the validation cohort.

**Table 4:** Clinical characteristics and CT findings for validation cohort

Features	HP masses (*n* = 16)	STs(*n* = 16)	*P* Value
Age(year)	43.56 ± 12.49	57.51 ± 7.49	0.001^a^
Location (the lower part/other)	12/4	1/15	< 0.001
Border (ill defined/ well defined)	10/6	4/12	0.033
Contour (flat or hill-like/other)	6/10	1/15	0.026
Enhancement grade (marked/ other)	13/3	2/14	0.001
CT enhancement value (> 27.50 HU/< 27.50 HU)	13/3	5/11	0.004
Relative enhancement ratio (> 0.66/< 0.66)	13/3	4/12	0.001
Relative enhancement ratio to pancreas (> 0.72/< 0.72)	13/3	6/10	0.012
LD/SD(> 1.29/< 1.29)	12/4	6/10	0.033

^a^*P* < 0.05. Calculated with Student’s t-test. Other calculated with χ2 test. *LD* long diameter, *SD* short diameter

## Discussion

This retrospective study included gastric lesions less than 3 cm in diameter; therefore, thin-layer images were required to analyze the CT features. Variables with significant differences in primary cohort were compared in validation cohort and there were significant differences in all above variables between two groups in validation cohort.

This study revealed significant differences in tumor location and border between the two groups, which were consistent with a previous report [20]. STs were more often located in the upper part (78%, 39/50), followed by the middle part (16%, 8/50), whereas most HP masses were located in the lower part (66%, 33/50), followed by the middle part (30%, 15/50). This trend of HP masses has been confirmed by many experts [21–23] and the hypothesis that HP masses are fragments separated from the main pancreas during the embryonic rotation process may explain this distribution [8, 24]. Most STs (90%, 45/50) exhibited well-defined margins, whereas most HP masses (56%, 28/50) exhibited ill-defined margins. These ill-defined margins were related to the histological structure of the HP masses. HP masses consisted of pancreatic acini, ductal components, and islets at different proportions. Approximately 27 to 76.6% of the HP masses exhibited prominent acinar features [14, 21] with lobular architecture. When this component was located in the peripheral part, the margin of the tumor was ill-defined [25]. However, the ill-defined margin was considered to be an adverse factor for the risk grading of STs in a previous study [26].

Significant differences were found in tumor contour and LD/SD ratio. In our study, 42% (21/50) of STs were round, but only 8% of (4/50) HP masses were round. Jang [27] also reported that other submucosal tumors are more likely to be round than HP masses (46.2% vs 6.7%). An HP mass is defined as ectopic flat glandular tissue with pancreatic acinar formation, and therefore commonly resembles the slender appearance of a normal pancreas and often exhibits a broad base on endoscopy [28]. HP masses of the mesentery are more elongated in shape than gastric HP masses, according to Seo [29]. In contrast, STs are real neoplasms, composed of spindle cells, epithelioid cells, or a mixture, with an obvious vertical growth trend. Our results showed that the mean SD for HP masses was significantly smaller than that for STs, but the LD did not differ significantly between the two groups. The greater LD/SD ratio also indicated a wall growth pattern for HP masses. In our study, the diagnostic value of the LD/SD ratio was medium, with an AUC of 0.707. An LD/SD ratio greater than 1.29 was found to be one of the critical imaging features of HP masses for differentiating it from STs. The LD/SD ratio trend was consistent with that mentioned in a previous study [14], but with a lower cut-off value. We concluded that the greater the diameter of an ectopic pancreatic mass, the more obvious the LD/SD ratio trend will be.

Highly significant differences were observed between the two groups in both qualitative and objective analyses. Qualitative analysis results indicated that both lesions presented homogeneous enhancement and had a greater enhancement grade in the venous phase than in the arterial phase. Hence, the CT attenuation values in the venous phase were used in subsequent calculations. In objective analysis, we assessed the enhancement grade by using the CT enhancement value and the enhancement ratio, rather than the values in the venous phase. The application of the enhancement value and ratio may reduce the influence of differences in machine and individual variations, which have been a factor in many previous studies [30, 31]. The presence of marked enhancement is a crucial finding with regard to avoiding misdiagnosis of HP masses and distinguishing them from STs. The study by Kim [14] indicated that the enhancement grade of HP masses has a close relationship with histological components. HP masses with predominant acini present greater enhancement than those with predominant ducts [22, 32]. Additionally, the enhancement ratio of tumor to pancreas is also crucial for distinguishing HP masses from STs. The average ratio of HP masses was significantly greater than that of STs and closer to 1, which is consistent with previous magnetic resonance imaging findings [27]. The AUC of the enhancement ratio to pancreas was greater than that of the enhancement value and the enhancement ratio, which indicated the ratio of the CT value of an HP mass to that of the pancreas is more valuable for identification than the variables (the enhancement value, the enhancement ratio) of the lesion itself.

In our study, both tumors predominantly exhibited an endo-luminal growth pattern; 30% of STs exhibited a predominantly exo-luminal or mixed growth pattern, which was lower than the proportion reported in a previous study on tumors less than 4 cm in diameter [14]. In addition, STs with a mean diameter of 10 cm also exhibit an obvious exo-luminal growth pattern [33]. We concluded that the larger a tumor is, the more obvious the exo-luminal growth pattern will be. The smaller a tumor is, the more difficult identification will be.

In our study, some findings, such as the presence of prominent thickness of the overlying mucosa, central umbilication, and multiple lesions, were detected in only a few (10, 6, 2%, respectively) patients with HP masses, and other findings, such as calcification and ulceration were detected in only a few (10, 8%, respectively) patients with STs. We assessed whether these findings constitute specific characteristics. First, central umbilication suggesting a rudimentary duct is present radiographically in only 16 to 25% of HP masses [14, 34], but present endoscopically in 35 to 60% of HP masses [12, 35]. Endoscopic ultrasonography is superior to CT in the assessment of mucosal surfaces. To our knowledge, no studies have reported STs with central umbilication, but ulceration may be confused with central umbilication, which was observed in 8% of STs in our study. Second, recurrent inflammatory changes caused by HP masses may explain the prominent thickness and enhancement of the overlying mucosa indicating microscopic gastritis in HP masses in a previous study [14]. Third, only one patient with HP exhibited multiple lesions; however, a previous study reported the presence of multiple lesions as a typical characteristic of succinate dehydrogenase deficient STs [36]. Therefore, the presence of multiple lesions is a rare and non-specific feature. Finally, HP with calcification has been reported [37], indicating that calcification is also not a specific feature.

Our study had several limitations. First, two CT scanners were used in our retrospective study, resulting in nonconformity of the scanned parameters and volumes of contrast media. However, we believe that morphological features may be unaffected by nonconformity, and the application of the enhancement values, enhancement ratios, and the enhancement ratios to pancreas may greatly reduce the influence of nonconformity. Second, histological analysis was not performed and radiologic-pathologic correlation was not assessed in our study because a complete pathological specimen was not available for all the included patients, and we could not guarantee that the sample and the ROI were at the same level. Third, HPs of mainly cystic composition were excluded. This induces a bias, but considering their particular CT findings the diagnosis of mainly cystic HP does not constitute a significant radiological problem. Finally, logistic regression analysis, texture analysis, and nomography [38] were not performed in this study. Larger prospective investigations are needed to confirm the present findings.

## Conclusions

In conclusion, HP has characteristic CT features for differentiating it from STs in the stomach. No significant differences were observed in growth patterns between the two lesions less than 3 cm in diameter. An LD/SD ratio greater than 1.29, an enhancement value greater than 27.50 HU, an enhancement ratio greater than 0.66, and an enhancement ratio to pancreas greater than 0.72 were critical CT features for differentiating HP masses from stromal tumors in our study.

## Abbreviations

AUC: The area under the curve
CI: 95% confidence intervals
CT: Computed tomography
HP: Heterotopic pancreatic
LD: Long diameter
ORs: Odds ratio
ROC: Receiver operating characteristic
SD: Short diameter
STs: Stromal tumors
